# Digital-Twin-Assisted Edge-Computing Resource Allocation Based on the Whale Optimization Algorithm

**DOI:** 10.3390/s22239546

**Published:** 2022-12-06

**Authors:** Shaoming Qiu, Jiancheng Zhao, Yana Lv, Jikun Dai, Fen Chen, Yahui Wang, Ao Li

**Affiliations:** Communication and Network Laboratory, Dalian University, Dalian 116622, China

**Keywords:** digital twin, edge computing, resource allocation, Internet of Things

## Abstract

With the rapid increase of smart Internet of Things (IoT) devices, edge networks generate a large number of computing tasks, which require edge-computing resource devices to complete the calculations. However, unreasonable edge-computing resource allocation suffers from high-power consumption and resource waste. Therefore, when user tasks are offloaded to the edge-computing system, reasonable resource allocation is an important issue. Thus, this paper proposes a digital-twin-(DT)-assisted edge-computing resource-allocation model and establishes a joint-optimization function of power consumption, delay, and unbalanced resource-allocation rate. Then, we develop a solution based on the improved whale optimization scheme. Specifically, we propose an improved whale optimization algorithm and design a greedy initialization strategy to improve the convergence speed for the DT-assisted edge-computing resource-allocation problem. Additionally, we redesign the whale search strategy to improve the allocation results. Several simulation experiments demonstrate that the improved whale optimization algorithm reduces the resource allocation and allocation objective function value, the power consumption, and the average resource allocation imbalance rate by 12.6%, 15.2%, and 15.6%, respectively. Overall, the power consumption with the assistance of the DT is reduced to 89.6% of the power required without DT assistance, thus, improving the efficiency of the edge-computing resource allocation.

## 1. Introduction

As the growth rate of cloud-computing power is gradually unable to meet the exponentially growing real-time data processing requirements, edge-computing technology has been proposed. Edge computing migrates computing task scheduling to a location close to the data source for execution, effectively improving the computing efficiency. However, due to limited edge-computing resources, new challenges have been created for resource-allocation optimization. At the same time, the edge-computing resource allocation scheme also affects the user experience. This paper mainly conducts research on edge-computing resource allocation. In this section, we introduce the motivation, related studies, and contributions.

### 1.1. Motivation

With the rapid increase of intelligent Internet of Things (IoT) devices, the edge network facing the physical world generates massive amounts of perception data. Nevertheless, cloud computing cannot support the low-latency computing requirements of these IoT devices. Therefore, edge computing has been proposed to reduce the transmission time between IoT devices [1,2,3]. The concept of edge computing is to move part of the computing load to the network edge closer to the user-end and employ edge nodes, e.g., base stations and switches, to complete the computing [4].

Currently, IoT and edge computing supports industrial, medical care, electric power, and agriculture applications, etc. [5,6,7]. However, due to the limited resources of edge devices, resource-efficient task allocation schemes must be developed under certain communication delay conditions. The current research on edge-computing resource allocation primarily considers the total energy consumption, battery power, computing load, and network load to optimize the resource allocation [8,9,10,11]. During edge-computing resource allocation for a single node, the number of applications to be served is considered, while, when allocating multiple nodes, problems, such as minimizing the energy consumption and load balancing, are primarily considered [12].

### 1.2. Related Studies

Intelligent optimization algorithms have been used to solve optimization problems in edge computing. For instance, Alfakih et al. delivered offloaded tasks to virtual machines in edge servers in a planned way to minimize the computing time and service costs. Additionally, the authors proposed an integrated accelerated particle swarm optimization scheme based on dynamic programming as a multi-objective (APSO) algorithm for dynamic task scheduling and load-balancing technology [13].

Luo et al. established an offloading communication and computing framework for vehicle edge computing. By jointly considering offloading decision-making and communication and computing resource allocation to minimize delay and cost, the edge resources can be efficiently scheduled. Indeed, a particle swarm optimization based on the computational offloading (PSOCO) algorithm was previously proposed [14].

Moreover, Subbaraj et al. used a local search strategy based on crowd search for resource allocation scheduling, which improved the allocation success rate [15]. Liu et al. proposed a joint-optimization objective to evaluate the unavailability level, communication delay, and resource waste and used a biogeographic optimization algorithm to solve the optimization objective [16].

The whale optimization algorithm has also been applied to solve the problem of edge computing and has effectively improved the edge-computing system’s efficiency. For instance, Huang et al. proposed a multi-objective whale optimization algorithm (MOWOA) based on time and energy consumption to solve the optimal offloading mechanism of computational offloading in mobile edge computing [17]. Xu et al. analyzed a three-layer network architecture for an Industrial Internet of Things (IIoT) that collaboratively and wirelessly powered an edge-computing network, using an improved hybrid whale optimization algorithm to maximize the efficiency of wireless sensor devices by offloading the decision-making, power allocation, computing resources, energy harvesting time, and residual energy [18].

A DT is defined as a virtual representation of a physical system, updated through the information exchange between the physical and virtual systems [19]. Currently, DT technology has been applied to assist edge computing [20,21,22,23,24] and provide a global perspective for edge computing task offloading and resource allocation by establishing a DT of physical entities [25]. Using DT technology reflects the operating status of the edge device and observes the errors of the DT and physical entities in real time, which are analyzed and calculated through the DT.

This strategy affords the unloading and the resource-allocation process to neglect real-time interactions between the user equipment and edge servers, improving the task efficiency and reducing the system energy consumption [26,27]. In [28], the authors developed a DT-enabled mobile edge computing (MEC) architecture. The delay minimization problem was proposed in the MEC model and was performed according to the transmission power, user association, intelligent task offloading, and DT-estimated CPU processing rate. This scheme solved and improved the performance delay problems.

Reference [29] built an edge-computing network combining DT and edge computing to improve the edge-computing network performance and reduce the resource overhead through DT technology. In [30], the authors established a dynamic DT of the air-assisted Internet of Vehicles and deployed a DT system in the UAV for unified resource scheduling and improved energy efficiency. A DT edge network was also suggested in [31], where the authors considered a DT of edge servers to evaluate the status of edge servers, improve the average offload delay, and reduce system costs.

### 1.3. Contributions

Although DT technology was initially applied in edge computing, the above research mainly considered delay and power consumption issues. Nevertheless, during the DT-assisted edge-computing resource allocation, improving the resource allocation balance can reduce the waste of resources. Thus, this paper uses DT to optimize the resource allocation of edge computing. In traditional edge-computing resource allocation, the assignment task must be transmitted to the edge server, which allocates resources according to the task.

However, the emergence of DT technology enables the edge server to have a resource device model so that the edge server can directly allocate tasks according to the resource device to reduce the overhead of the transmission process. Therefore, we use DT technology to assist edge-computing servers in resource allocation; establish a joint-optimization function for transmission delay, resource allocation imbalance rate, and power consumption; and develop a method based on the whale optimization algorithm. The main contributions of this work are as follows:

(1) We consider a DT-assisted edge-computing resource-allocation system and establish a DT-assisted resource-allocation model.
(2) We build a joint-optimization model of time, energy consumption, and resource allocation imbalance rate through the DT and the physical model.
(3) We improve the whale optimization algorithm to solve the DT-assisted edge-computing resource-allocation optimization problem.

The remainder of this paper is organized as follows. Section 2 describes the system model and elaborates on the resource-allocation problem of DT-assisted edge computing. Section 3 introduces the proposed improved algorithm, and Section 4 presents the simulation and comparison results. Finally, Section 5 concludes this paper.

## 2. System and Computation Model

The system comprises two parts, a physical entity and its DT. The physical entity comprises the users, edge servers, and resource devices. The DT is deployed inside the edge server, and, after the client generates the task parameter model, it reaches the edge server. The DT in the edge server allocates resources to the task according to the energy consumption, storage, and computing, generates the optimal result, and transmits the results to the user device. The user equipment directly transmits the task to the resource equipment, which completes the calculations. The DT cannot entirely and accurately simulate the physical entity as there will be specific errors, and the cumulative error will increase with time. Therefore, parameter calibration between the physical entity and the DT is performed at regular intervals in the system. For ease of understanding, Table 1 lists the key notations in this paper.

### 2.1. DT-Assisted Edge-Computing Model

**Figure 1:**
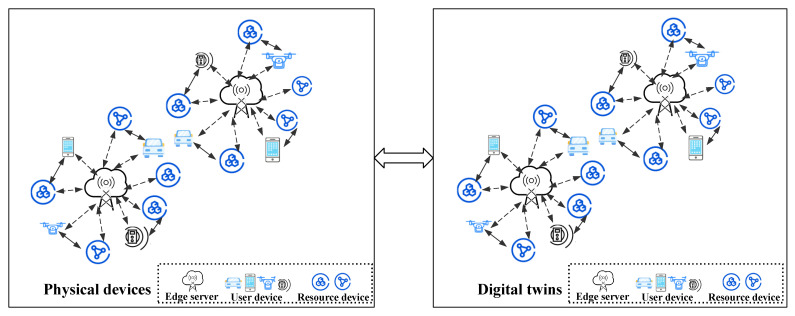
DT-assisted edge-computing resource-allocation model.

As illustrated in Figure 1, a DT-assisted edge-computing setup consists of user devices, edge servers, resource devices, and DT. The user equipment initiates a task request to the edge server, the edge device allocates computing resources to the requested task, and the DT is deployed in the edge server.

Let U={1,2,3,…,u} represent user equipment that needs to request resources and let E={1,2,3,…,e} denote edge servers. The resource devices for allocation managed by each edge server are given by S={1,2,3,…,s}. The DT of the resource devices in the DT layer provides the real-time resource usage and is represented by $DT^{s}=\left\{s_{1},s_{2},s_{3},\ldots ,s_{n}\right\}$, where $s_{i}=\left\{q_{1},q_{2},q_{3},\ldots ,q_{m}\right\}$, and $q_{i}$ is a particular resource of $s_{i}$. A task model in an edge server is represented as $J=\{j_{1},j_{2},j_{3},\ldots ,j_{l}\}$, where the *i*-th task is $j_{i}=\left\{z_{1},z_{2},z_{3},\ldots ,z_{m}\right\}$, and $z_{i}$ is the demand quantity of a task for a specific resource. According to the resource situation of the resource device’s DT, the DT deployed in the edge server selects the optimal allocation strategy for each task request and assigns the task to the physical resource device to complete the calculation.

### 2.2. DT-Assisted Edge-Computing Latency Model

Edge computing brings computing closer to the user to reduce latency. Therefore, during the edge-computing resource allocation, the processing delay is a vital evaluation index, mainly divided into the DT model’s communication, computation, and correction delay. The DT model is not calibrated continuously and is updated only when the difference between the DT model and the physical entity reaches a threshold. We use $T^{0}$ to denote its update time. The simulated transmission time ${\tilde{T}}_{m}^{\tau }$ of the *m*-th task is defined as:

(1) $${\tilde{T}}_{m}^{\tau }=\frac{D_{m}}{B_{m}}$$

where $D_{m}$ is the data volume of the *m*-th task simulated by the DT, and $B_{m}$ is the bandwidth used by the *m*-th task of the DT. Since the DT model and the physical entity are not the same, the error $\Delta T_{m}^{\tau }$ of the simulated transmission of the *m*-th task is defined as:

(2) $$\Delta T_{m}^{\tau }=\frac{D_{m}+\Delta D_{m}}{B_{m}+\Delta B_{m}}-\frac{D_{m}}{B_{m}}=\frac{B_{m}\Delta D_{m}-\Delta B_{m}D_{m}}{B_{m}\left(B_{m}+\Delta B_{m}\right)}$$

where $\Delta D_{m}$ is the DT’s data error providing analog transmission, and $\Delta B_{m}$ is the bandwidth error of the analog transmission. Hence, the total time $T^{\tau }$ of the task data transfer is defined as:

(3) $$T^{\tau }=\sum_{e\in E}\sum_{j\in J}\left({\tilde{T}}_{e,j}^{\tau }+\Delta T_{e,j}^{\tau }\right)$$

The task calculation time of the DT simulation is represented by ${\tilde{T}}_{m}^{c}$, which can be calculated by Formula (4).

(4) $${\tilde{T}}_{m}^{c}=\frac{C_{m}}{f_{n}}$$

where $C_{m}$ is the calculation amount of the *m*-th task of the DT simulation and $f_{m}$ is the calculation frequency allocated by the resource device for the *m*-th task. Therefore, the calculation time error $\Delta T_{m}^{c}$ generated by the DT is defined as:

(5) $$\Delta T_{m}^{c}=\frac{C_{m}+\Delta C_{m}}{f_{m}+\Delta f_{m}}-\frac{C_{m}}{f_{m}}=\frac{f_{m}\Delta C_{m}-\Delta f_{m}C_{m}}{f_{m}\left(f_{m}+\Delta f_{m}\right)}$$

where $\Delta C_{m}$ is the calculated error of DT providing the analog transmission and $\Delta f_{m}$ is the error between the actual computing frequency of the *m*-th task and the digital twin simulation computing frequency. Then, the total computation time $T^{c}$ of all tasks is defined as:

(6) $$T^{c}=\sum_{e\in E}\sum_{j\in J}\left({\tilde{T}}_{e,j}^{c}+\Delta T_{e,j}^{c}\right)$$

Therefore, the total time for data transfer, computation, and DT model correction during the allocation process is represented by $T_{tot}$, which can be calculated using Formula (7).

(7) $$T_{tot}=\sum_{e\in E}\sum_{j\in J}\left({\tilde{T}}_{e,j}^{\tau }+\Delta T_{e,j}^{\tau }\right)+\sum_{e\in E}\sum_{j\in J}\left({\tilde{T}}_{e,j}^{c}+\Delta T_{e,j}^{c}\right)+\sum_{e\in E}T_{e}^{0}$$

### 2.3. DT-Assisted Edge-Computing Energy-Consumption Model

The battery power of edge-resource devices is limited, thereby, affecting the task-allocation efficiency considering the power usage and maximizing the use of edge resources. Therefore, this paper establishes the following power consumption model, with the power consumption of the *m*-th task defined as:

(8) $$P_{m}=k{f_{m}}^{3}t=k{\left(f_{m}+\Delta f_{m}\right)}^{2}\left(C_{m}+\Delta C_{m}\right)$$

where *k* is the coefficient of different computing resources related to the physical device chip, $f_{m}$ is the calculation frequency of the *m*-th task, and *t* is the calculation time required by the *m*-th task. The total computing power consumption for all computing tasks is:

(9) $$P_{tot=}\sum_{e\in E}\sum_{j\in J}k{\left(f_{e,j}+\Delta f_{e,j}\right)}^{2}\left(C_{e,j}+\Delta C_{e,j}\right)$$

### 2.4. A Balanced Model of Edge-Computing Resource Allocation Assisted by DT

We consider the balance of different resource allocations in the resource devices during resource allocation. If a particular resource type occupies too much space, it will no longer be possible to allocate resources, and many other resources may be left, thereby, reducing the actual resource usage rate of the devices. Therefore, during resource allocation, balancing the resource allocation is mandatory. When the difference between the remaining resources in different dimensions during resource allocation is large, most resources are wasted, and thus the resource utilization rate is lower. Assuming that there are *N* resource devices, the resource in the *m*-th resource device is $S_{m}$, the total number of resource types is *M*, and the simulated resource usage rate is ${\tilde{U}}_{i}^{m}$. Then, we use $U_{i}^{m}$ to represent the real usage rate, which can be defined as:

(10) $$U_{i}^{m}={\tilde{U}}_{i}^{m}+\Delta U_{i}^{m}$$

where $\Delta U_{i}^{m}$ is the error between the usage rate of the *i*-th resource of the *m*-th device simulated by the digital twin and the real usage rate. Then, the average utilization rate of *M* resources of the *m*-th device is represented by $U_{m}^{avg}$, which is defined as:

(11) $$U_{m}^{avg}=\frac{\sum_{i\in M}U_{i}^{m}}{M}$$

Then, the resource allocation imbalance rate of the *m*-th device is represented by $D_{m}$, which is defined as:

(12) $$D_{m}=\sqrt{\frac{1}{M}\sum_{i\in M}{\left(U_{i}^{m}-U_{i}^{avg}\right)}^{2}}$$

The imbalance rate of the resource allocation for all resource equipment is represented by $\bar{D}$, which is defined as:

(13) $$\bar{D}=\frac{\sum_{e\in E}\sum_{s\in S}D_{e,s}}{N}$$

When the physical device decides that the task must be handed over to the edge resource to complete the calculation during the calculation process, these tasks will be transferred to the edge computing device to complete the calculation. This paper obtains the status of the edge-resource devices through the DT model deployed on the edge server and assigns tasks through the computing task model.

Finally, the assignment results are distributed to the user devices, which directly transmit the tasks to the resource devices to complete the calculations. During task assignment, assigning tasks nearby can reduce the transmission delay; however, such an assignment may cause resource waste due to unbalanced resource utilization. Therefore, we comprehensively consider the delay, power consumption, and resource utilization and establish a DT auxiliary system for optimal resource allocation by minimizing the delay, power consumption, and resource utilization. Finally, the target problem optimized in this paper is expressed as follows:

(14) $$\begin{array}{cc} \; & \min W=\theta_{1}T_{tot}+\theta_{2}P_{tot}+\theta_{3}\bar{D}\\ \; & s.t.\;\;\;C1:0<\theta_{1},\theta_{2},\theta_{3}<1\\ \; & \;\;\;\;\;\;\;\;C2:\theta_{1}+\theta_{2}+\theta_{3}=1\\ \; & \;\;\;\;\;\;\;\;C3:\sum_{j\in J}P^{i,j}<P_{\max}^{i},\forall i\in E\\ \; & \;\;\;\;\;\;\;\;C4:\sum_{j\in J}f^{i,j}<f_{\max}^{i},\forall i\in E\\ \; & \;\;\;\;\;\;\;\;C5:T^{i,j}<T_{\max}^{i},\forall j\in J,\forall i\in E \end{array}$$

where θ is the weight coefficient, C3 is the constraint on the power consumption of edge-resource devices, C4 is the constraint on the frequency of resource devices, and C5 is the constraint on the task time.

## 3. Improved Whale Optimization Algorithm

### 3.1. Whale Optimization Algorithm

The whale optimization algorithm (WOA) [32] is a meta-heuristic intelligent optimization algorithm proposed by Mirjalili et al., which considers three behaviors by simulating humpback hunting: surround, search, and attack.

#### 3.1.1. Surrounding the Prey

Humpback whales identify the prey’s location and surround it. The whale individual closest to the prey is the current optimal solution, and the other whales approach the current optimal individual. The position update formula is as follows:

(15) $$D=\left|C\cdot X^{*}\left(t\right)-X\left(t\right)\right|$$

(16) $$X\left(t+1\right)=X^{*}\left(t\right)-A\times D$$

where *t* is the number of iterations, $X^{*}\left(t\right)$ is the current optimal whale individual position vector, and $X\left(t\right)$ is the current whale individual position vector. *A* and *C* are coefficients, which are calculated as follows:

(17) A=2a×r−a

(18) C=2·r

where *a* decreases linearly from 2 to 0 during the iterative process, and *r* is a random number within [0,1].

#### 3.1.2. Bubble Attack

During updating the position, the humpback whale spits out bubbles surrounding the prey and hunts in a spiral manner with a certain probability, defined as:

(19) $$X\left(t\right)=D\cdot e^{bl}\cdot \cos\left(2\pi l\right)+X^{*}\left(t\right)$$

where *b* is a constant and *l* is a random number within [−1,1].

#### 3.1.3. Searching for Prey

When $\left|A\right|>1$, the whale stays away from the current optimal whale individual and performs a global search, unaffected by the currently optimal whale. The position update formula is as follows:

(20) $$D=\;\left|C\times X_{rand}-X\left(t\right)\right|$$

(21) $$X\left(t+1\right)\;=\;X_{rand}-A\times D$$

where $X_{rand}$ is the location of a random individual whale.

### 3.2. DT-Assisted Edge-Computing Resource Allocation Based on the Improved Whale Optimization Algorithm

The whale optimization algorithm has a convergence speed in the process of resource allocation, and it is easy to fall into the problem of local optimal solutions. Therefore, when solving this problem, we improved the whale optimization algorithm and proposed IPWOA. First, we coded the computing tasks so that the whale optimization algorithm can solve the resource allocation tasks. After that, in order to improve the early convergence speed of the whale optimization algorithm, we proposed a greedy initialization strategy.

Through the effective initialization of the whale population, the initial whale population is in a better state. Finally, we redesigned the whale’s predation and search strategy. By updating the optimal value of the whale’s own search process in combination with individual whales, the local search effect of the whale optimization algorithm is improved. The following is the specific improvement strategy.

#### 3.2.1. Encoding

During the application, we encode optimized tasks and map resource devices to whale populations. Here, the distance of the whale population is the difference between different resource devices—that is, the distance between resource devices. The coded index of the population represents the tasks assigned by generations, and its length is the same as the number of tasks. The filled parameter is the number of the selected edge-computing resource, and the corresponding number indicates the resource code.

**Figure 2:**
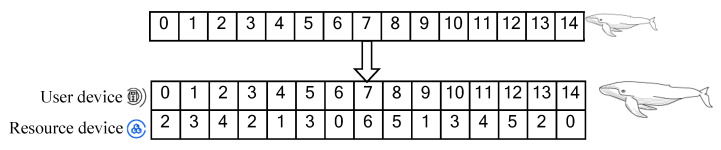
Resource equipment and user task encoding method.

The coding format is illustrated in Figure 2, where the number of 0–14 in the population is the number of an optimization task, and the resource number is 0–6. Figure 2 depicts the task assignment, where the assignment of task No. 0 is calculated by resource device No. 2.

#### 3.2.2. Greedy Initialization Method

The initialized population significantly influences the results during the algorithm’s execution, and a better-initialized population speeds up the algorithm’s convergence. Hence, we designed an initialization method based on a greedy strategy according to the optimization problem, and the obtained initialization population is a locally optimal solution. For the initialization algorithm, we employed formula (22) to allocate a resource device for each task, which is the resource device with the minimum value of the optimization objective function generated in the resource device set. For example, in Figure 2, the optimal solution corresponding to task 0 is first calculated to be 2, and, based on this, the optimal solution for the other tasks is solved. The corresponding allocation scheme is an optimal local solution, considered to be an initialized population.

Simultaneously, the assignment results from different tasks may differ, as, in the beginning, an initial assignment task index is randomly generated to select where to start the assignment task to ensure population diversity.

(22) $$X_{i}^{t}=\min\left\{w_{1},w_{2},w_{3},\ldots ,w_{n}\right\}$$

where $X_{i}^{t}$ is the computing resource allocated by the *i*-th task, and $w_{n}$ is the cost value generated by the resource device allocated by the corresponding task.

#### 3.2.3. Improve Optimization Methods

A Nonlinear convergence factor.
  The convergence factor of the original whale optimization algorithm is linear; however, the optimization algorithm must quickly enter the local search stage after greedy initialization. The linear convergence factor is given by formula (23), and the optimization algorithm quickly enters the local search stage.
  (23) $$a=2\cdot (1-\sqrt{\frac{t}{t_{\max}}})$$
B Improving the predation mechanism.
  When the whale population is preying, the whales will approach the prey; however, this will force the whale population to fall into the optimal local solution. Therefore, to rebalance the global and local optimizations, a self-learning item is designed and defined as:
  (24) $$\begin{array}{cc} \; & Y\left(t+1\right)=Y\left(t\right)+Ar_{1}\cdot \left(X^{\alpha }-X\left(t\right)\right)+vr_{2}\cdot \left(X^{*}-X\left(t\right)\right)\\ \; & X(t+1)=X(t)+Y(t+1) \end{array}$$
  where $X^{\alpha }$ is the optimal value of the current whale individual, $r_{1}$ and $r_{2}$ are random numbers, and *v* is an inertia weight.
C Improving the siege mechanism.
  A spiral surrounds the original whale. In this paper, the individual whales have an updated position depending on the latest position of the spiral to avoid falling into a locally optimal solution: the global and local optimizations as well as the self-learning item are designed and defined as:
  (25) $$\begin{array}{cc} \; & X^{\delta }=D\cdot e^{bl}\cdot \cos\left(2\pi l\right)+X^{*}\left(t\right)\\ \; & Y\left(t+1\right)=Y\left(t\right)+Ar_{1}\cdot \left(X^{\alpha }-X^{\delta }\right)+vr_{2}\cdot \left(X^{*}-X\left(t\right)\right)\\ \; & X(t+1)=X(t)+Y(t+1) \end{array}$$
  where $X^{\delta }$ is the individual whale after the current spiral update position.

The pseudo-code for the improved algorithm (Algorithm 1) is provided below:
**Algorithm 1:**IPWOA**Input:** tasks *J*, computing resources *S*, $t_{\max}$**Output:** BestAllocation1: Set t=0, set number of tasks tasksNum, set $t_{\max}$, searchNo, allocation plan *P*;2: Initial each allocation plan *P* using Equation (22);3: Calculate the fitness of each allocation plan *P* using Equation (14);4: Best allocation bP = the best allocation plan;5: While $t<t_{\max}$6:    Update *a*, *A*, *C*, *l*, *p*;7:    For i=0 to tasksNum:8:       if p<0.59:          if $\left|A\right|<1$10:             Update resource allocation plan *P* using Equation (24);11:          else12:             Update resource allocation plan *P* using Equation (20), Equation(21);13:       else14:          if $\left|A\right|<1$15:             Update resource allocation plan *P* using Equation (25);16:          else17:             Update resource allocation plan *P* using Equation (19);18:    Check if any distribution plan the search space and amend it;19:    Calculate the fitness of each distribution plan using Equation (14);20:    Update the best allocation bP if there is a better solution;21:    t=t+1;22: return best allocation bP

**Figure 3:**
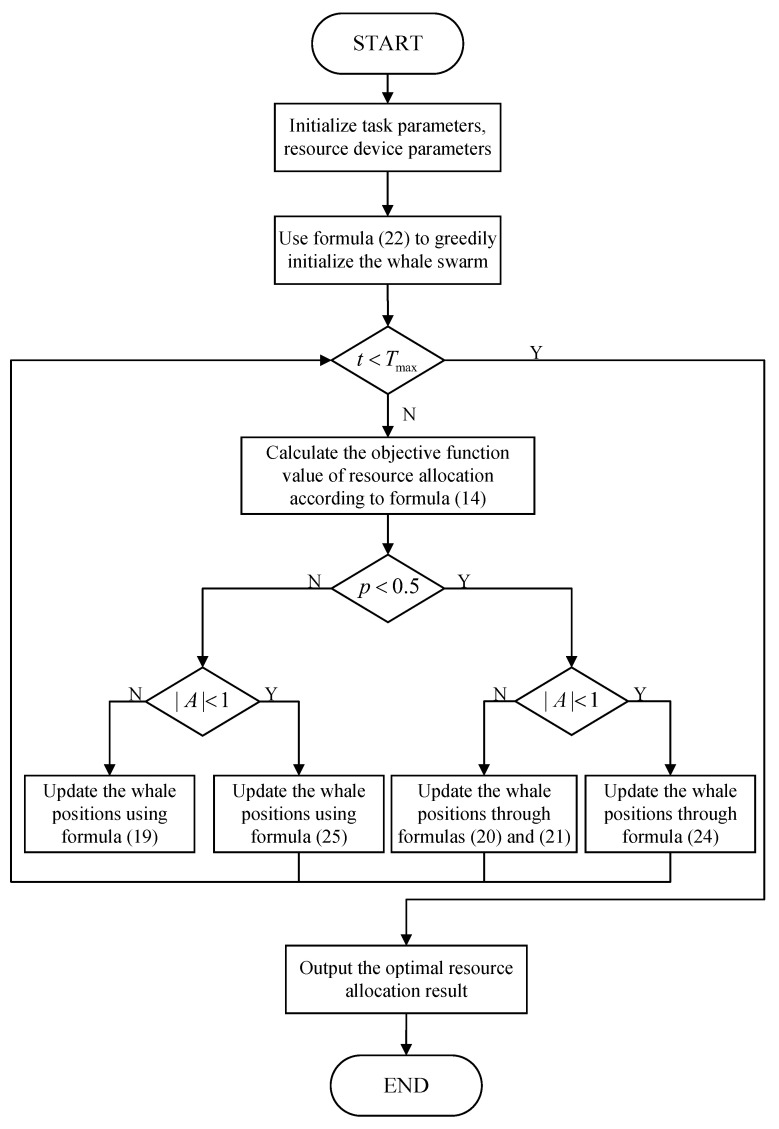
IPWOA flow chart.

The IPWOA solution task assignment flow chart is illustrated in Figure 3, comprising the following major steps:

Step 1: Initialize the parameters, such as the memory, computing resources, and time constraints of edge devices and computing tasks. Initialize the parameters of the whale population. Set basic parameters, such as the number of whale populations and the maximum number of iterations.

Step 2: Use formula (22) to greedily initialize the whale swarm to generate a set of initial optimal solutions.

Step 3: If the termination iteration condition is reached, step 7 is performed. Otherwise, step 4 is performed.

Step 4: Calculate the objective function value of resource allocation according to formula (14), and record the optimal individual in the current whale group and the global optimal whale individual. Then, generate a random number *p*. If p<0.5, go to step 5. Otherwise, go to step 6.

Step 5: Generate a random number *A*. If $\left|A\right|<1$, update the whale positions through formula (24). Otherwise, update the whale positions through formulas (20) and (21), and repeat step 3.

Step 6: Generate a random number *A*. If $\left|A\right|<1$, update the whale positions using formula (25). Otherwise, update the whale positions using formula (19) and repeat step 3.

Step 7: Output the optimal whale individual and record the optimal computing resource allocation result.

## 4. Simulation and Result

In this section, we build simulation experiments to evaluate the proposed model and solution method. The simulation experiments were performed on a computer configured with an Intel Core I7-7700HQ 2.8 GHz CPU and 16 GB RAM.

### 4.1. Simulation Setup

For the simulated experiments, we consider that the resources possessed by the resource devices may be different. The computing frequency range of the simulated resource devices is [50, 80] kHz, the bandwidth resources are [180, 230] KB, and the memory resources are [190, 250] MB. The error is in the range of (0,1), and the range of coefficient k is (0, 1). The computing resources occupied by the corresponding different tasks are [8, 13] kHz, the memory resources are [18, 25] KB, and the occupied memory is [8, 13] kHz. The resources are [15, 26] MB, and the time constraint is [0.7, 1.25] s. The simulation experiments are conducted by utilizing the above parameters.

### 4.2. Impact of the Improved Algorithm on Resource Allocation

**Figure 4:**
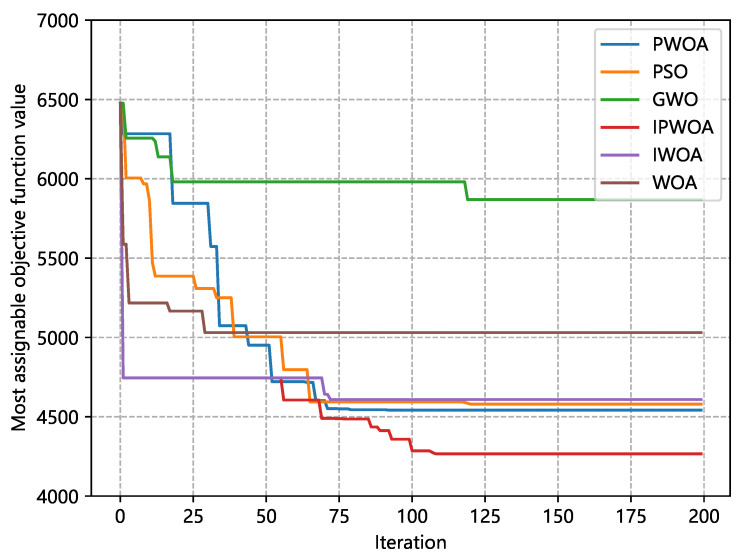
Optimized objective values after multiple iterations of different algorithms. Each algorithm iterates 200 times.

As illustrated in Figure 4, we compare the iterative results of different algorithms for resource-allocation problems. Specifically, the simulation experiments involve the particle swarm optimization algorithm (PSO), gray wolf optimization algorithm (GWO), whale optimization algorithm (WOA), the initialized improved whale optimization algorithm (IWOA), the improved whale optimization algorithm in Section 3.2.3 (PWOA), and the proposed improved whale optimization algorithm (IPWOA). All algorithms were applied for 200 iterations to solve the optimization task. Since the original WOA was randomly initialized, the early convergence was slower than in the IPWOA. Furthermore, by improving WOA, the optimization results were better than WOA and the other algorithms, with the experimental results demonstrating that IPWOA attained an optimal result that was 15.2% smaller than the original WOA.

**Figure 5:**
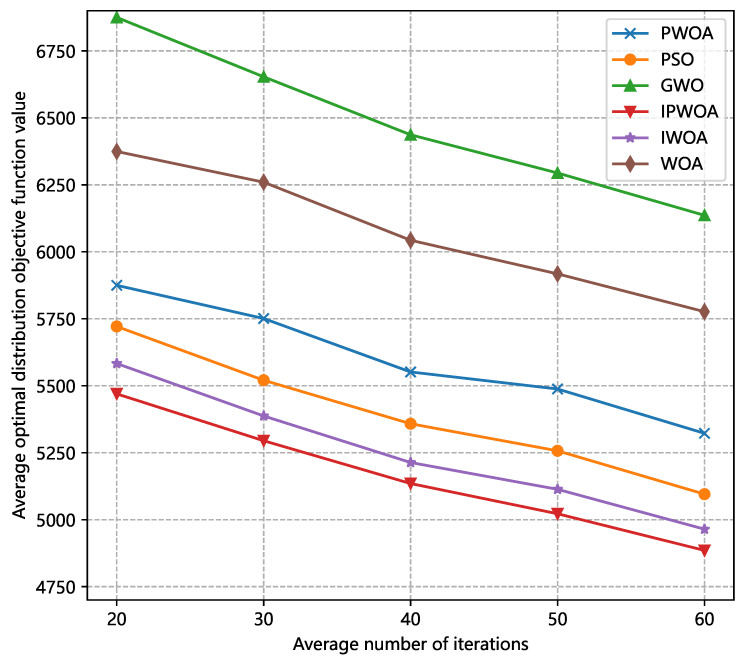
The optimization target value after multiple rounds of iterations of different algorithms. Each algorithm was executed for multiple rounds, each round of iterations included 200 times, and the average optimal optimization target value was generated.

Additionally, this paper analyzed the algorithm’s average optimal results over multiple iterations to verify that the improvement effect is more generic. We conducted 60 rounds of experiments on each algorithm, and each experiment was iterated 200 times. We recorded the average optimization target values at 20, 30, 40, 50, and 60 rounds. The corresponding results are reported in Table 2 and Figure 5. The average result of 60 iterations shows that the optimal result of IPWOA was 12.6% higher than that of WOA, and it also achieved better results compared with PSO and GWO.

**Figure 6:**
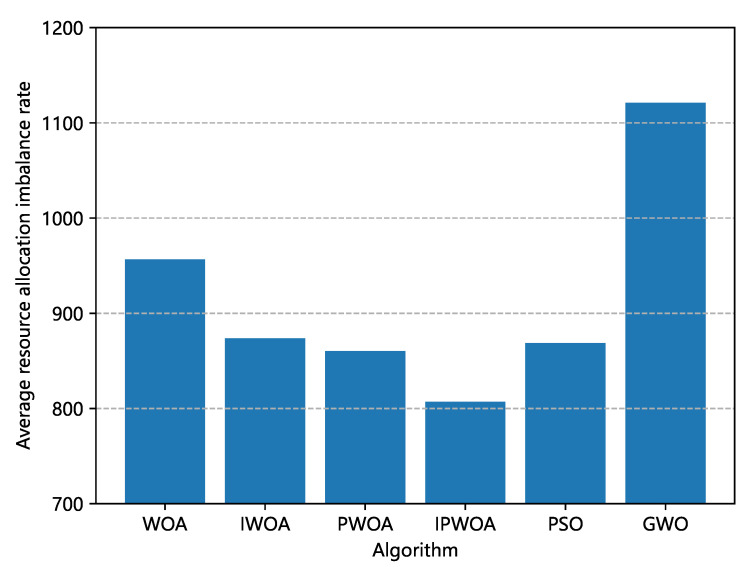
Comparison of the power consumption of resource devices generated by different algorithms. The resource device power consumption was generated by the optimal allocation scheme of each algorithm.

**Figure 7:**
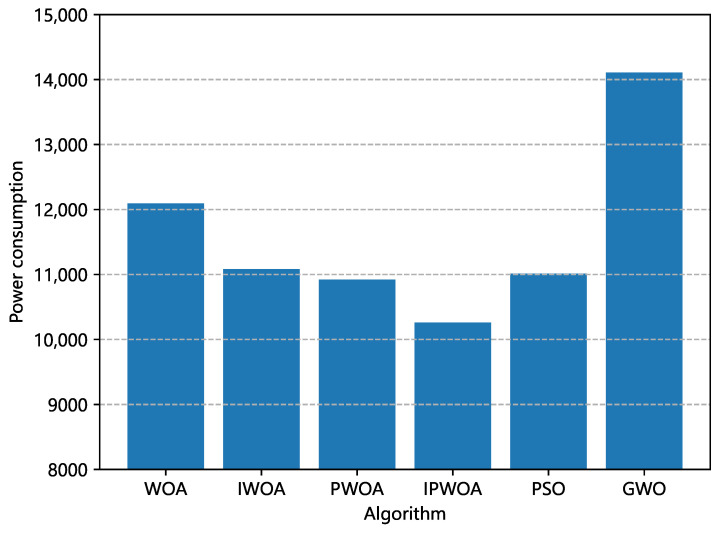
Comparison of the power consumption impact of different algorithms for edge computing. The resource device power consumption was generated by the optimal allocation scheme of each algorithm.

We also analyzed the final optimal allocation scheme obtained by each optimization algorithm. Figure 6 and Figure 7 are comparisons of the unbalanced rates of resource allocation and power consumption brought about by the optimal allocation scheme of each algorithm. Figure 6 shows that, in the allocation scheme of the optimal solution, the power consumption of IPWOA was reduced by 15.2% compared with WOA, and Figure 7 shows that the average resource allocation imbalance rate was reduced by 15.6%.

### 4.3. Optimization of an Edge-Computing Resource-Allocation System with a DT System

The DT technology can assist in allocating edge-computing resources, allowing the edge-computing system to reduce the number of data transmissions, and affording resource devices to devote more time to computing. When the resource device has more computing time, the allocated frequency is relatively less, and the computing power consumption is reduced.

**Figure 8:**
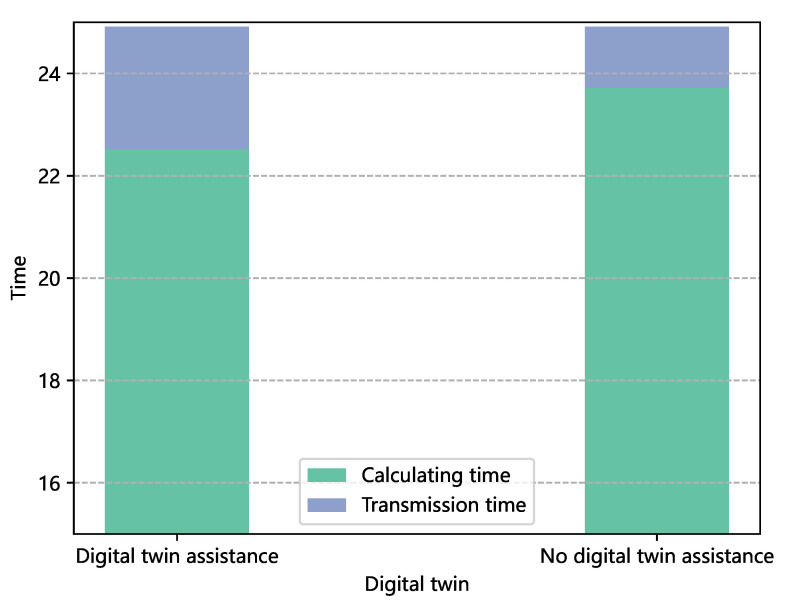
Comparison of the impacts of a DT on the task-computing time.

**Figure 9:**
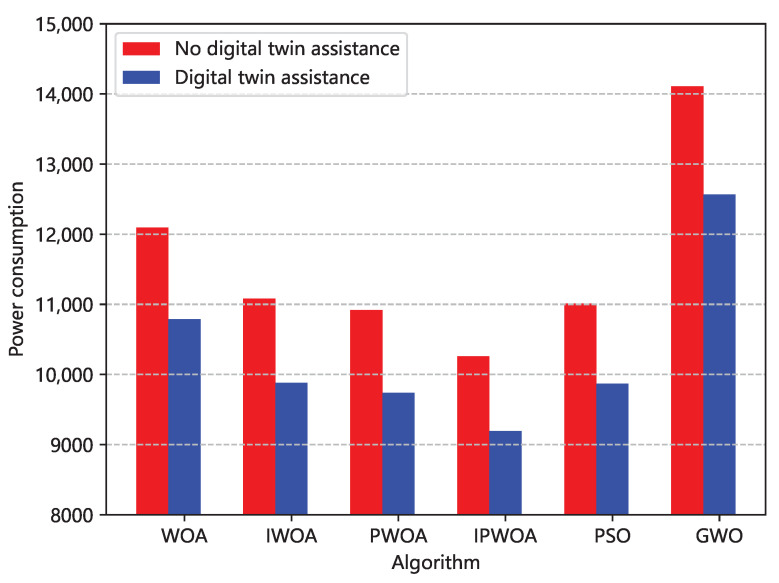
Comparison of the impacts of a DT on the edge-computing power consumption. The resource device power consumption was generated by the optimal allocation scheme of each algorithm.

Figure 8 highlights that, by optimally allocating the resources obtained by the improved whale under the same time and time constraints, exploiting the DT assistance increases the proportion of computing time over the total time. Therefore, we further analyzed the impact of digital twin assistance on resource-allocation power consumption in terms of the time saved. As illustrated in Figure 9, the power consumption with the assistance of the DT was 89.6% of the one not assisted by a DT.

## 5. Conclusions

With the development of edge-computing technology, IoT applications are increasingly connected to edge-computing systems, which improves the efficiency of edge-computing resource allocation and improves the user experience.

In this paper, a DT-assisted edge-computing resource-allocation model was developed to solve the problem of low resources and high-power consumption in the process of edge-computing resource allocation. Specifically, we considered: (1) Through digital-twin-assisted edge computing, a problem model of power consumption, delay, and an unbalanced resource-allocation rate was established. (2) A greedy initialization strategy was proposed, which effectively improved the early convergence speed of the whale optimization algorithm. (3) An improved search strategy was proposed, and the accuracy of the algorithm search was improved by introducing the optimal item of the individual whale search process.

Simulation experiments showed that the IPWOA reduced the resource allocation and allocation objective function value, the power consumption, and the average resource allocation imbalance rate by 12.6%, 15.2%, and 15.6%, respectively. The power consumption with the assistance of the DT was reduced to 89.6% of the power required without DT assistance.

In our future work, we will further consider the impacts of dynamic user devices on the optimization problem and continue to study the problem of digital technology offloading tasks for dynamic user edge computing.

## Figures and Tables

**Table 1:** Key notations.

Notation	Description
*U*	set of users who need to request computing resources
*E*	set of edge server
*S*	set of resource devices
$DT^{s}$	DT set of resource devices
$s_{i}$	set of resources of the *i*-th resource device, $s_{i}\in DT^{s}$
*J*	set of computing tasks
$j_{i}$	resources required for the *i*-th task, $j_{i}\in J$
$z_{k}$	demand of the *i*-th task on the *k*-th resource, $z_{k}\in j_{i}$
$D_{m}$	data volume of the *m*-th task simulated by the DT, $D_{m}\in j$
$B_{m}$	bandwidth used by the *m*-th task of the DT simulation
${\tilde{T}}_{m}^{\tau }$	simulated transmission time of the *m*-th task
$\Delta D_{m}$	error of the *m*-th task data volume of the DT simulation
$\Delta B_{m}$	error of the transmission bandwidth of the *m*-th task simulated by the digital twin
$\Delta T_{m}^{\tau }$	transit time error for the *m*-th task of a DT simulation
${\tilde{T}}_{m}^{c}$	transit time for the *m*-th task of the DT simulation
$C_{m}$	computational amount of the *m*-th task of the DT simulation
$f_{m}$	calculation frequency assigned by the resource device to the *m*-th task
$\Delta C_{m}$	calculated error of DT providing the analog transmission
$\Delta f_{m}$	error between the actual computing frequency of the *m*-th task and the digital twin simulation computing frequency
$\Delta T_{m}^{c}$	computational time error for the *m*-th task of a DT simulation
$P_{m}$	computational energy consumption generated by the *m*-th task
$U_{i}^{m}$	actual usage rate of the *i*-th resource of the *m*-th resource device
${\tilde{U}}_{i}^{m}$	usage rate of the *i*-th resource of the *m*-th resource device simulated by the DT
$\Delta U_{i}^{m}$	error of the utilization rate of the *i*-th resource of the *m*-th resource device simulated by the DT
$U_{m}^{avg}$	average resource usage rate of the *m*-th resource device
$D_{m}$	unbalanced resource-allocation rate of the *m*-th resource device

**Table 2:** Average optimization objective value after multiple iterations of different algorithms.

Algorithm	Round 20	Round 30	Round 40	Round 50	Round 60
PWOA	5917.58	5768.95	5554.72	5453.48	5284.10
PSO	5540.77	5365.33	5174.71	5050.52	4918.83
GWO	6778.41	6657.15	6435.48	6283.25	6111.39
IPWOA	5463.13	5266.74	5080.25	4982.79	4846.93
IWOA	5575.54	5384.47	5206.87	5109.37	4957.43
WOA	6102.32	5978.89	5757.57	5674.87	5542.64

## Data Availability

Not applicable.
